# The effect of terminal impedance on aortic morphology and hemodynamics: an *in vitro* phantom study using flow field visualization

**DOI:** 10.3389/fbioe.2023.1175916

**Published:** 2023-04-24

**Authors:** Huimin Chen, Wenjun Wang, Dengji Liu, Zhen Cao, Yi Yang, Ying He, Qingzhuo Chi

**Affiliations:** ^1^ Key Laboratory of Ocean Energy Utilization and Energy Conservation of Ministry of Education, School of Energy and Power Engineering, Dalian University of Technology, Dalian, China; ^2^ Department of Cardiovascular Surgery, Dalian Municipal Central Hospital, Dalian, China; ^3^ Department of Neurosurgery, The First Affiliated Hospital of Dalian Medical University, Dalian, China

**Keywords:** aortic dissection, *in vitro*, silicone phantom, terminal impedance, particle image velocimetry

## Abstract

To investigate the risk factors for aortic dissection tearing, we fabricated a simplified patient-specific aortic silicone phantom using the brush-spin-coating method. The aortic phantom only includes the aorta from the ascending aorta to the descending aorta, without other branches. We designed two experiments to investigate the alteration of aortic morphology and intravascular hemodynamics using the particle image velocimetry method. The results revealed dilation and elongation of the aortic phantom, especially the ascending aorta, after the phantom’s terminal resistance was increased. Additionally, the particle image velocimetry results demonstrated an increased vortex region, which caused the inner side of the aortic wall to become scoured by blood. This study suggests that the deformation of the inner side aortic wall and the change in hemodynamics in response to the increased terminal resistance may be a risk factor for aortic tearing and should be monitored.

## 1 Introduction

Aortic dissection is a high-mortality disease that manifests as a separation of the middle layer from the inner layer caused by leaking blood, forming a false lumen. Long-term abnormal hemodynamics might weaken the interlaminar adhesion strength of the aortic wall, decrease the elastic component of the aortic wall, and gradually deepen the fibrosis of the intimal layer, increasing the possibility of tearing (Loscalzo et al., 2018). However, the occurrence of aortic dissection still needs a trigger, e.g., an increase in inter-aortic pressure caused by a physical impact (Elefteriades, 2008; Ziganshin et al., 2019). Therefore, a better understanding and assessment of aortic morphology and hemodynamics is needed, especially with regard to increased blood pressure, to reveal the risk factors of tearing.

The *in vitro* model is a widely used tool for studying complex hemodynamics and aortic morphology. Burriesci et al. (2020) produced a model comprising a rigid wall lumen and an elastic-wall silicone tube by three-dimensionally printing a dissolvable personalized inner core. Using this method, a rigid wall lumen containing a false lumen was also fabricated (Burriesci et al., 2020). Marconi et al. (2018) used an ideal aortic dissection phantom model with a tear and a false lumen in development and opportunistic intervention research of type B aortic dissection. They pointed out that the developmental behavior of acute type B aortic dissection is very complex. Bonfanti et al., 2017 measured the intraluminal velocity and pressure in a true and false lumens in an elastic aortic dissection phantom. The measured data from the simulation were consistent with the *in vivo* data. In a follow-up study, intraluminal aortic deformation and wave propagation during aortic dissection were investigated further (Bonfanti et al., 2018). Recently, an aortic phantom fabrication method, called the brush-spin-coating method, was designed (Chi et al., 2021a). This technology has been used to study many cardiovascular diseases, aortic dissection (Chi et al., 2022), arterial stenosis (Mu et al., 2022), and so on.

To assess the hemodynamics of a phantom *in vitro*, the particle image velocimetry (PIV) method is widely used in flow field visualization. Yip et al., 2011 used PIV to assess the effects of aortic compliance; Salameh et al. (2019) provide some insight into the hemodynamics and perfusion of radiological contrast in patent and non-patent aortic dissection models obtained using PIV, revealing the relationship between the location and size of an entry tear and the elevation of hemodynamic loading, as well as the risk of tearing; Zadrazil et al. (2020) further investigated the hemodynamics of ideal aortic dissection with different entry tear sizes using the PIV method and validated their results using computational fluid dynamics simulations. The hemodynamics in a patient-specific aortic phantom were recently investigated using PIV; this suggests that the experimental approach can be used not only to validate the results of computational fluid dynamics but also for surgical training and procedural planning (Franzetti et al., 2022).

To investigate aortic morphology and hemodynamics as blood pressure increases, we conducted *in vitro* experiments on a simplified patient-specific aortic silicone phantom, fabricated by our patented technology, using PIV to visualize the flow field. In this study, we first explain how the silicone material is chosen and how to manufacture and calibrate the aortic phantom. Then, the setup of the *in vitro* pressurization experiment and PIV experiment are described. In the Results section, we present the results of the experiments. We then discuss the clinical implications of this study as well as the limitations and future work. Finally, we draw some conclusions.

## 2 Materials and methods

### 2.1 Selection of silicone material based on human aortic tissue

The tissues of the aortic wall are composed of elastin fibers and collagen fibers: elastin fibers are more compliant, while collagen fibers are stiffer, resulting in a non-linear constitutive behavior of the aortic wall, as revealed by a multistage stress–strain curve. A widely used constitutive framework of the aortic wall is the Holzapfel–Gasser–Ogden material model, in which the collagen fibers are considered as contributing an additional anisotropy to the constitutive law (Holzapfel et al., 2000). Tensile testing of aortic wall tissues reveals that at first only the elastic fibers are strained, while the collagen fibers interspersed between the tissues remain in a bent or folded state. In other words, in a situation of very small strain, only the elastin fibers bear a uniaxial load. In this situation, the tissues exhibit a linear stress–strain response (Raghavan et al., 1996). As the tension increases, some of the less-wrinkled collagen fibers gradually become taut. As revealed by Samila and Carter (Samila and Carter, 1981), the slope of the stress–strain curve increases with the increase in total stiffness. At the final stage, all collagen fibers are involved in tensile bearing, and the total stiffness reaches a maximum. The stress–strain curve becomes linear again until the tissues have all yielded.

**FIGURE 1:**
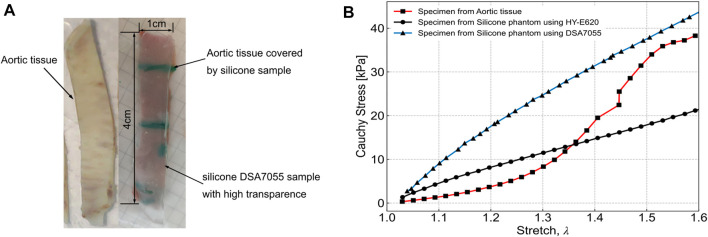
Tensile testing specimen and results from Chi et al., 2022. **(A)** Left: aortic tissue. Right: silicone sample made with DSA7055, which is highly transparent, and the aortic tissue sample underneath. Both samples were the same size (4 cm × 1 cm). **(B)** Tensile testing results of three samples: aortic tissue, HY-E620 silicone, and DSA7055 silicone.

In our experiments, two factors determined the selection of silicone. First, the mechanical properties of the phantom should be as similar as possible to aortic wall tissue. Second, the refractive indexes of the phantom and of the working fluid should be the same, for better visualization during the PIV. Therefore, we conducted tensile testing using two types of silicone phantom and one sample of aortic wall tissue. The two types of silicone were HY-E620 (Hong Ye E620, Shen Zhen Hong Ye Jie Technology Co Ltd., Shen Zhen, China) and DSA7055 (DSA 7055, Guangdong Doneson New Materials, Guang-dong, China). For the tensile testing, the three samples were cut into 4 cm × 1 cm rectangular pieces, following the work of Raghavan et al. (1996), as shown in Figure 1A, and the samples were tested under uniaxial loading. The tensile testing results were published previously (Chi et al., 2022) and are shown in Figure 1B. From the results, the curve for silicone DSA7055 is closer to the aortic tissue than that of silicone HY-E620 when the stretch reaches 1.5. Meanwhile, the DSA7055 silicone has a more suitable refractive index than HY-E620 silicone. In other words, the refractive index of DSA7055 silicone is more similar to that of the working fluid, which is a 1:1 mixture of glycerin and 0.9% normal saline. As Figure 1A shows, the DSA7055 silicone sample is transparent enough to see the aortic tissue under it. Thus, DSA7055 silicone was used to manufacture the phantom.

### 2.2 Construction of the *In Vitro* test system

#### 2.2.1 Manufacture of the aortic phantom and its calibration

**FIGURE 2:**
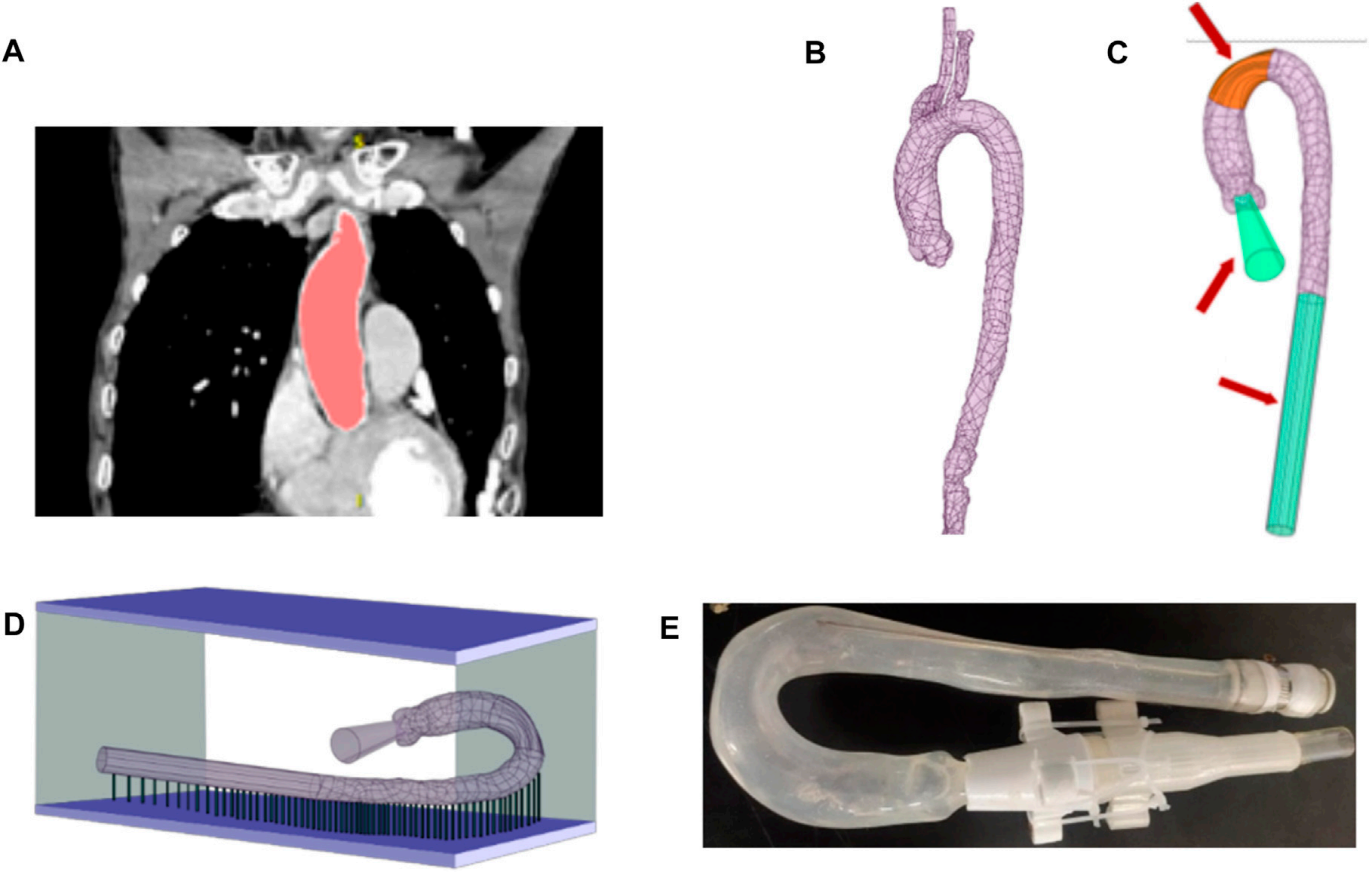
Process of fabricating the phantom. **(A)** Extraction of the shape and size of the aorta from a computed tomography image using Simpleware™ Scan IP. **(B)** Reconstructed aorta model. **(C)** Simplification of the model by removing branches at the position marked in orange and adding auxiliary inlets and outlets, marked in green. **(D)** Fabrication of the model in a three-dimensional printer. **(E)** The finished aortic phantom, made using the brush-spin-coating method.

To manufacture the aortic phantom, we reconstructed a three-dimensional aortic model from a computed tomography image using Simpleware™ Scan IP software, as shown in Figures 2A, B (ethical use protocols were complied with when using the computed tomography image). The model was simplified to contain only the main aorta, from the ascending aorta to the descending aorta, omitting all other branches and adding auxiliary inlets and outlets, as shown in Figure 2C. Then, a stereolithographic file of the model was exported to a three-dimensional printer. The three-dimensional printing process is shown in Figure 2D. Finally, the aortic phantom, shown in Figure 2E, was made using the brush-spin-coating method. The process of fabricating the phantom using the brush-spin-coating method is described in detail in our previous study (Chi et al., 2021a). Using the brush-spin-coating method, we can obtain a phantom with a uniform wall thickness (Chi et al., 2021a) of 1.8 mm.

**FIGURE 3:**
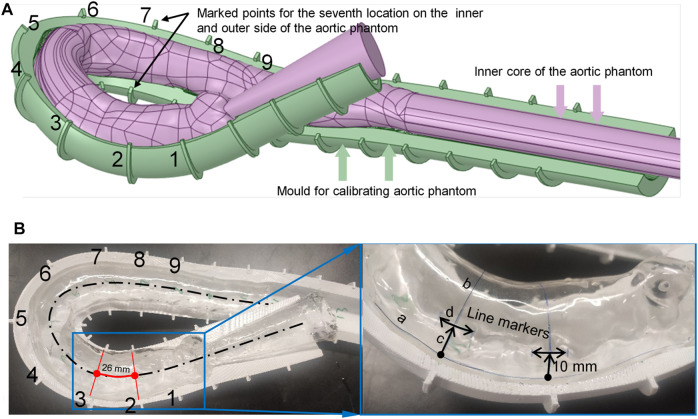
Method for marking calibration lines on the surface of the aortic phantom. **(A)** The aortic model and assisted calibration mold in Ansys SpaceClaim. **(B)** Nine drawn line markers partition the aorta into eight segments.

To measure the morphological alterations of the aortic silicone phantom, the outer surface of the silicone phantom should be calibrated before the experiment. The model was placed in a calibrated mold, which was made using the expansion function in Ansys SpaceClaim, and a cut plane was created along the centerline of the aorta by applying the sweep function. The cut plane was applied to split the external aortic mold into top and bottom halves. Figure 3A shows the bottom half of the mold (the green model) after splitting.

The mold needs some markers to calibrate the phantom. Along the centerline of the aorta, nine equidistant cross-sections intersect the calibration mold, marked as points 1 to 9 (shown in Figure 3A), where the pink color indicates the aortic model and the dark green model indicates the calibration mold. The calibration points at the seventh location are indicated in Figure 3A. The numbers are marked on the outside curve of the aorta; the opposite side is the inside curve of the aorta.

There are two types of marking line on the surface of the silicone in the model: axial marking lines along the centerline and radial marking lines perpendicular to the center line. The axial marking lines, for instance curve a–c marked on the outside of the model, are drawn on the surface of the phantom along the edge of the mold (both outside and inside the aortic phantom). These marks are used to measure the elongation ratio between the outside and inside of the aortic phantom. The radial marker lines are obtained by connecting the inner and outer marked points (the nine cross-sections intersect the surface of the aorta at the marked lines, e.g., line b–c in the right panel of Figure 3B), and are used to measure the dilation of the aortic phantom. These radial line markers divide the aortic model into eight segments, as shown in Figure 3B. Finally, the line segment markers (e.g., d in the right panel of Figure 3B), with a length of 10 mm, are made at positions that are 10 mm away from the outer marked points, and are used to measure the elongation of the aortic phantom on the outside. The markers are perpendicular to the connection line b–c. At each end of each of the marked 10 mm lines, short lines, perpendicular to the 10 mm marked lines, are drawn to assist in measuring the marked 10 mm lines at different pressures. As ink does not easily adhere to the silicone surface, a thin layer of silicone was applied to the surface of the marked aortic phantom to protect the marked lines.

#### 2.2.2 Setup of the *In vitro* pressurization experiment

**FIGURE 4:**
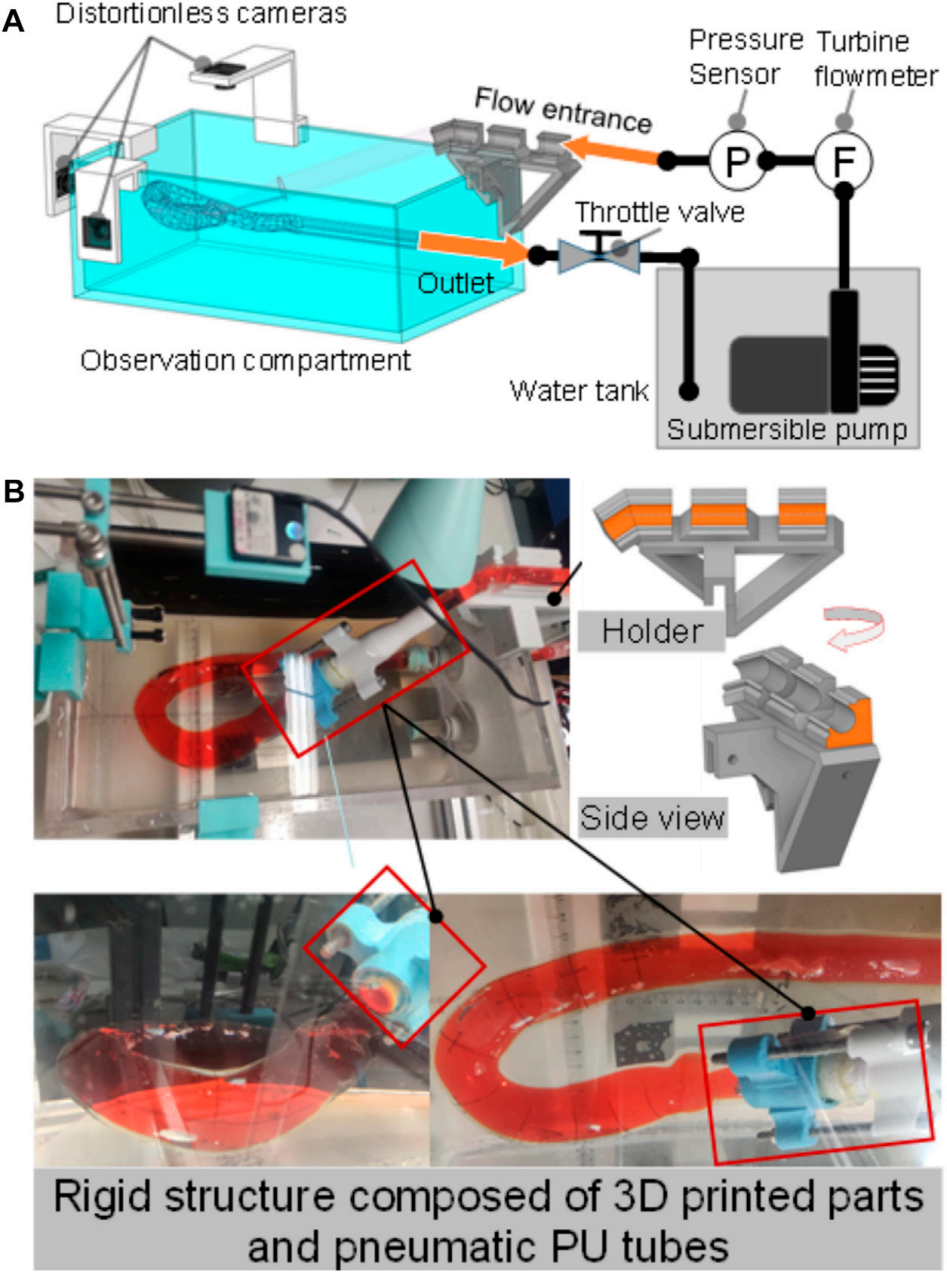
*In vitro* pressurization experiment. **(A)** Experiment setup, consisting of a submersible pump, a water tank, an observation compartment, a distortionless cameras, an aortic phantom, a throttle valve, a pressure sensor, and a turbine flowmeter. **(B)** Rigid structure composed of three-dimensionally printed parts and pneumatic polyurethane (PU) tubes to fix the inlet; the outlet is fixed to the cavity.

The *in vitro* experiment set up consists of a submersible pump (PT-6500, Shenzhen Laoyujiang Industrial Co., Ltd, Shenzhen, China), a pressure sensor, a turbine flowmeter, the aortic phantom, three distortionless cameras, a throttle valve, and a water tank, as shown in Figure 4A. In this setup, the aortic phantom is fixed rigidly at the inlet and outlet, as shown in Figure 4B. Except for the experimental section of the aortic phantom, the front and rear ends of the model are connected by hard structures to ensure the rigidity of the structure. At the front end of the aorta, there is a rigid pipeline composed of three-dimensionally printed parts and a pneumatic polyurethane tube. The rear end of the model is fixed to the observation cavity. The submersible pump is the power source for circulating the fluid through the phantom, and pressure and flow rate signals are recorded at a frequency of 10 Hz. The aortic phantom is immersed in working fluid in the observation compartment. The working fluid in the aortic phantom is the same as that in the observation compartment and is a 1:1 mixture of glycerin and 0.9% normal saline, the refractive index of which is one of the factors that influenced the choice of silicone material, as mentioned previously. The only difference is that the working fluid in the aortic phantom is stained with orange dye for the convenient measurement of the aortic contour, as shown in Figure 4B. The circulating fluid flows from the pump through the aortic phantom and then returns to the water tank. The throttle valve is used to adjust the intravascular pressure. Finally, the morphological alteration of the aortic phantom in response to the pressure change is recorded using the distortionless cameras.

The average cardiac output is approximately 5.2 L/min and the peak flow rate can reach 24.9 L/min during the systolic period. Because the model aorta used in the study has been simplified by removing all other branch vessels, the flow rate is adjusted to 6.5 L/min. To control the experimental variables, the steady flow experiment is conducted by maintaining the flow rate at 6.5 L/min and increasing the terminal resistance by adjusting the throttle valve, which indeed results in an increase in intravascular pressure. However, the reduced throttle port results in a change in the flow rate. Thus, we need to adjust the power of the pump to keep it stable at 6.5 L/min.

It is known that the maximum pressure in the aorta can be 38.7 kPa; this is observed during vigorous exercise and is an increase of approximately 22.7 kPa relative to the typical maximum human blood pressure (Blazek et al., 2019). Therefore, we keep the inlet flow rate constant at 6.5 L/min and increase the terminal resistance, resulting in a pressure change from 2.5 kPa to 24.30 kPa at the inlet. The aorta begins to dilate at a pressure of 3.25 kPa. Therefore, we start to record the morphology of the phantom when this point is reached.

#### 2.2.3 Flow field visualization studies

**FIGURE 5:**
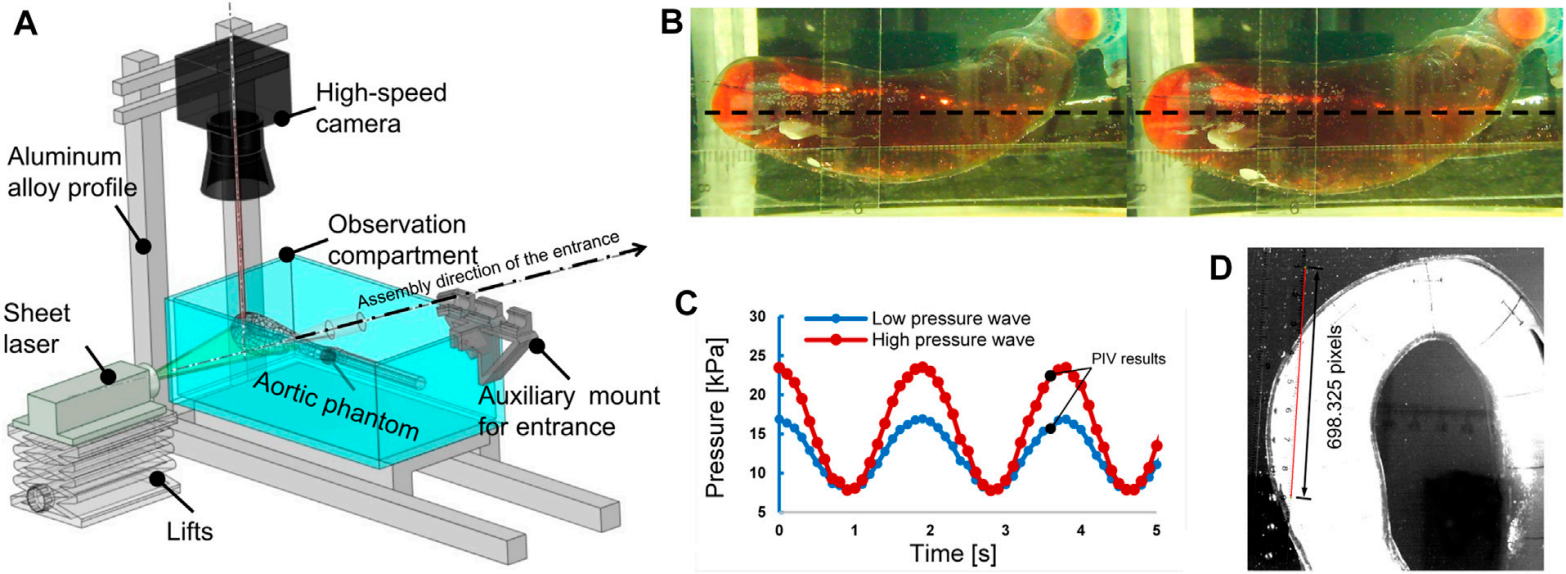
The particle image velocimetry (PIV) flow visualization experiment. **(A)** Layout of the experimental system for flow visualization. **(B)** Plane of laser irradiation (black dotted line). Left: pressure, 3.26 kPa. Right: pressure, 24.30 kPa. **(C)** Pulsating inlet pressure at different end impedances; the recording of PIV results began at the times marked with black points. **(D)** Calibration image of PIV.

To study the *in vitro* hemodynamics, the flow field was visualized using PIV. The experimental configuration remained the same as the pressurization experiments described in the previous section, but the distortionless cameras were replaced with a high-speed camera (Fastcam mini UX50, Photron, Japan), and a laser source (MGL-W-532, Changchun Institute of Optics., Ji Lin, China) was added, as shown in Figure 5A. The high-speed camera was set vertically over the experiment bench. The plane of the laser irradiation is shown in Figure 5B. Polyamide resin particles (100063-2, Beiting Measurement Technology (Beijing) Co., Ltd, China), with a diameter of 20 μm, were chosen as a tracer. Isopropanol solution was used to disperse the tracer particles and was poured gradually into the water tank after the tracer particles were wetted. Finally, the working fluid temperature was adjusted to 35 °C so that it was similar to the human core temperature, and the viscosity of the working fluid at that temperature was 4.5 × 10^−3^ kg/(m s). Refractive index tests showed that the phantom immersed in the working fluid produced a low black-and-white block aberration.

**FIGURE 6:**
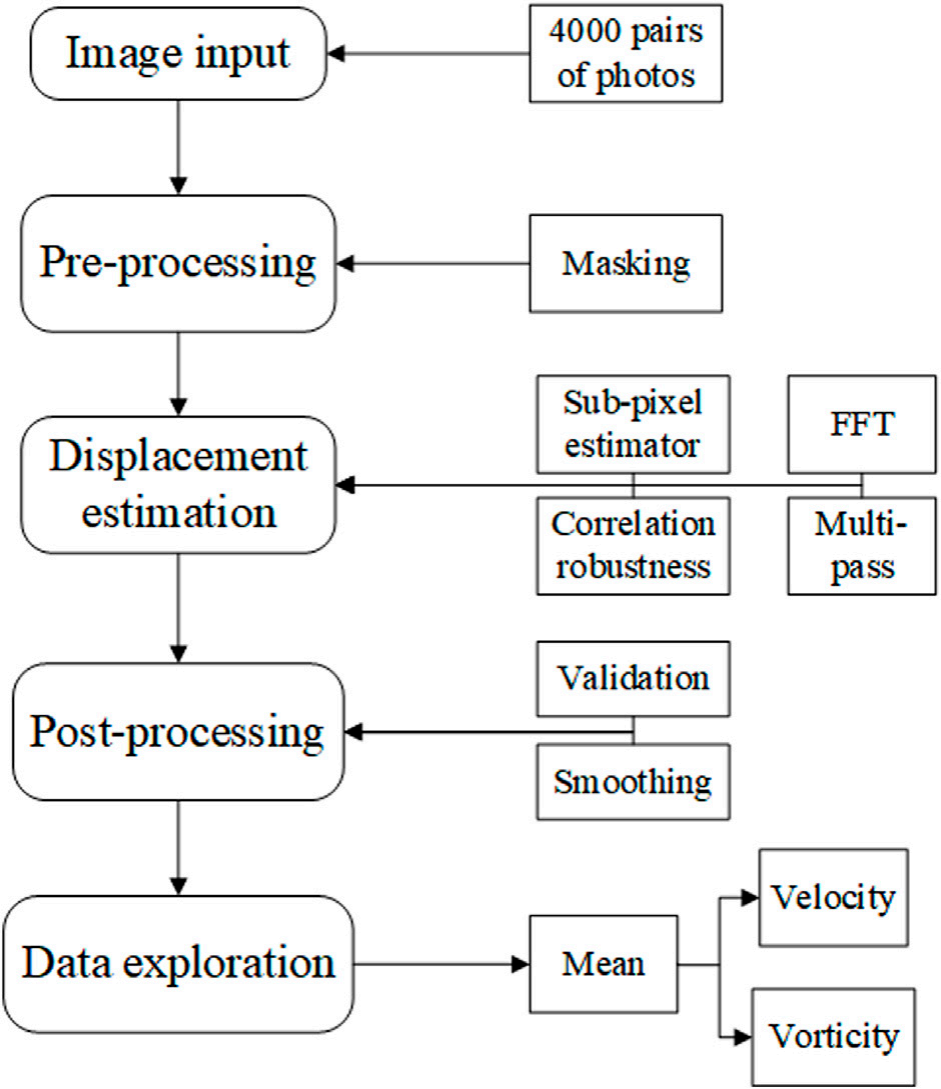
Particle image velocimetry process flow chart. FFT, fast Fourier transform.

Two types of inlet pressure profile, provided by the pump, low and high (as shown in Figure 5C), were used in the experiment. The pressure was increased by elevating the terminal impedance. To obtain a fully developed fluid field, the fluid was allowed to circulate through the apparatus for 10 min before photographs were taken with the high-speed camera. The frame rate was 2,500 frames per second, and the camera was operated for 2.4 s to obtain 6,000 photographs. A total of 4,001 photographs were chosen for analysis, combined into 4,000 pairs as photographs 1 and 2, photographs 2 and 3, *etc.* There were 4,000 pairs of photographs for each of the low- and high-pressure analyses. The calibration is shown in Figure 5D at a scale ratio of 8 cm/698.325 pixels; each pixel was measured in a Photron FASTCAM Viewer 4 by selecting two points on a ruler: 1 cm and 9 cm. Finally, the images were processed using the MATLAB-based open-source PIV post-processing package PIVlab, as shown in Figure 6 (Thielicke and Sonntag, 2021).

Using PIVlab, the 4,000 pairs of photographs were input to the program. Next, the photographs were masked to obscure the region outside areas 1 to 4, to focus on the hemodynamics in the ascending aorta and aortic arch. Then, the displacements between the pairs of photographs were estimated using the PIV algorithm of fast Fourier transform window deformation, multipass using two passes (pass 1 with an interrogation area of 64 and 32 steps and pass 2 with an interrogation area of 32 and 16 steps) a sub-pixel estimator, using Gauss 2 × 3-point, and standard correlation robustness. After this, the velocity was converted to real dimensions using the ratio 8 cm/698.325 pixels shown in Figure 5D, which was validated by removing velocities that were too large or too small, and smoothed at a low level. Finally, the velocity was calculated by averaging the velocity results of 4,000 pairs of photographs. More details of the algorithm of PIV and setting of PIVlab can be found in (Thielicke and Sonntag, 2021).

## 3 Results

### 3.1 Morphological changes of the aorta

**FIGURE 7:**
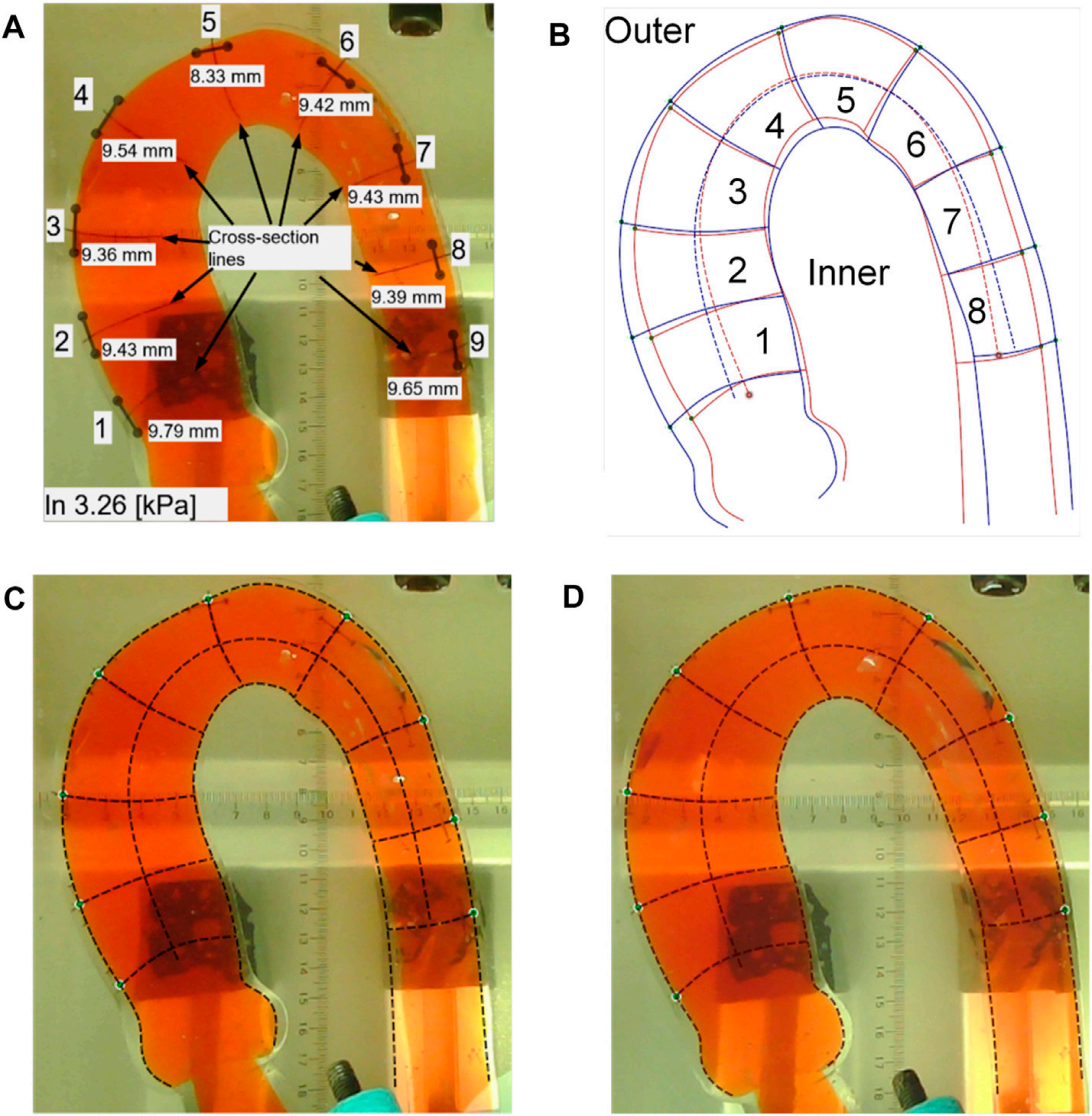
Processing results in the aortic phantom pressurization experiment. **(A)** The positions of the 10-mm length marker lines and the corresponding nine cross-section lines and the measured values of the 10-mm marker lines. **(B)** Changes in the location of the aortic subdivision contours before and after impedance increase. **(C)** Aortic profile extraction at a low pressure of 3.26 kPa. **(D)** Aortic contour extraction under a high pressure of 24.30 kPa.

**FIGURE 8:**
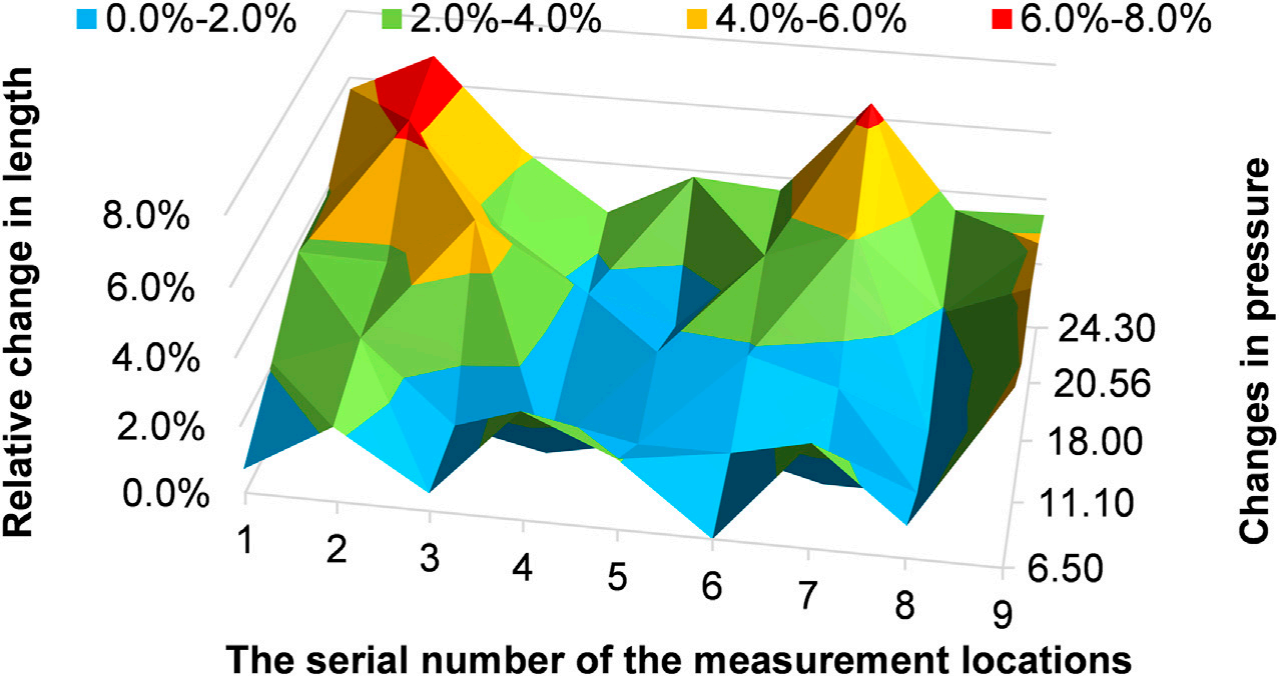
Relative length change of the 10-mm imprint line.

Two morphological changes in the aorta were recorded: elongation and dilation of the aortic phantom. To determine the elongation of the phantom, we measured the lengths of the 10-mm marker lines at the nine positions three times using a Vernier caliper (line segment d in Figure 3B; Figure 7A); the results of the length change as a function of pressure increase are shown in Figure 8. In addition, Figures 7C, D present the difference in elongation between the outside and inside curves of the aorta. Finally, the dilation of the aorta is represented by the length of the nine cross-section lines, corresponding to the positions of the 10-mm marked lines (the line b–c in Figure 3B and the cross-section lines indicated in Figure 7A).

For the 10-mm marked line, we measured its length at pressures of 3.26 kPa, 6.50 kPa, 11.10 kPa, 18.00 kPa, 20.56 kPa, and 24.30 kPa. Then we calculated the relative length changes $R_{i,j}$ as

$$R_{i,j}=\frac{l_{10mm,i,j}-l_{10mm,\min,j}}{l_{10mm,\min,j}}\times 100\%$$

where $l_{10mm,\min}$ is the length of the marked line at the lowest pressure (3.26 kPa), $l_{10mm,i}$ (*i* = 1, 2, 3, 4, 5) is the length of the marked line at pressures of 6.50 kPa, 11.10 kPa, 18.00 kPa, 20.56 kPa, and 24.30 kPa, respectively, and *j* = 1, 2, 3, 4, 5, 6, 7, 8, 9 is the position of the relevant 10-mm marked line. The results are shown in Figure 8.

As shown in Figure 8, 10-mm marked lines 2 and 7 have the maximal relative length change, and the relative length change can reach 6.9% of 10-mm marked line 2 at a pressure of 24.30 kPa. In particular, 10-mm marked lines 1 to 3 clearly elongate before 10-mm marked line 7 at a pressure of 11.10 kPa.

We measured the outside and inside contours and the cross-section lines in two situations: low and high pressure. At low pressure, we took photographs when the pressure reached 3.25 kPa, 3.26 kPa, and 3.95 kPa, as shown in Figure 7C. At high pressure, photographs of the aortic phantom were taken when the pressures were 20.56 kPa, 21.5 kPa, and 24.30 kPa, as shown in Figure 7D. Finally, the photographs were input to Ansys SpaceClaim to measure the length of the contours of the eight areas and nine cross-section lines.

**FIGURE 9:**
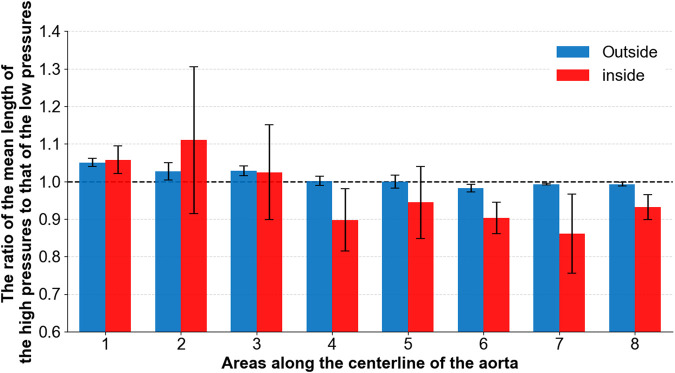
Ratio of mean length of the contour at high pressures to that at low pressures.

To determine the morphological differences of the outside and inside curves of the phantom after a pressure increase, we calculated the ratio of the mean length of the contour at high pressure (20.56 kPa, 21.5 kPa, and 24.30 kPa) to that at low pressure (3.25 kPa, 3.26 kPa, and 3.95 kPa). The ratios of the eight areas are shown in Figure 9. The blue bars indicate the ratio of the outside contour, while the red bars indicate the ratio of the inside contour. The results shown in Figure 9 reveal that the outside and inside of areas 1 to 3 elongate but the inside contour of areas 5 to 8 shortens. In particular, the inside curve of area 2 increases, while the inside curve of areas 4 and 7 is mostly reduced.

**FIGURE 10:**
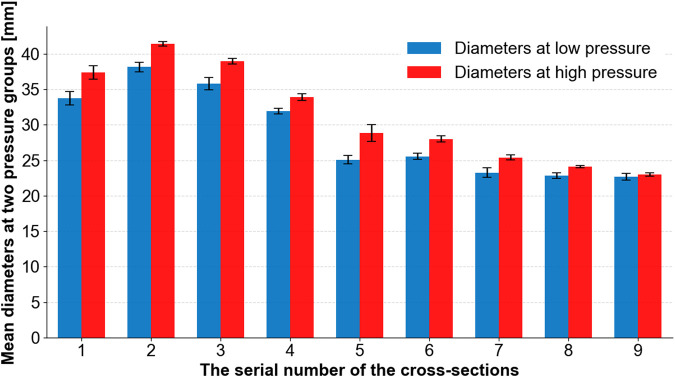
Mean diameters at two groups of pressures (low and high) at different cross-section lines.

The diameters of the aorta are represented by the lengths of the cross-section lines, which are used to indicate the dilation of the aorta. The results are shown in Figure 10, in which the blue bars indicate the diameters observed at low pressures (3.25 kPa, 3.26 kPa, and 3.95 kPa) and the red bars indicate the diameters observed at high pressures (20.56 kPa, 21.5 kPa, and 24.30 kPa). Figure 10 shows that the aorta dilates after a pressure increase. The cross-section line 9 only dilates slightly.

### 3.2 Fluid field Visualization in the aortic phantom by PIV

**FIGURE 11:**
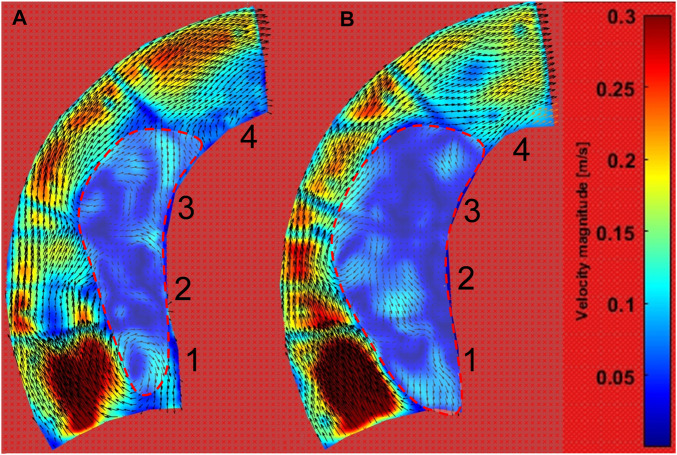
Velocity contour of the ascending aortic flow field at pulsating pressure. **(A)** The contour during low-pressure inlet conditions (blue line in Figure 5C). **(B)** The contour during high-pressure inlet conditions (red line in Figure 5C).

**FIGURE 12:**
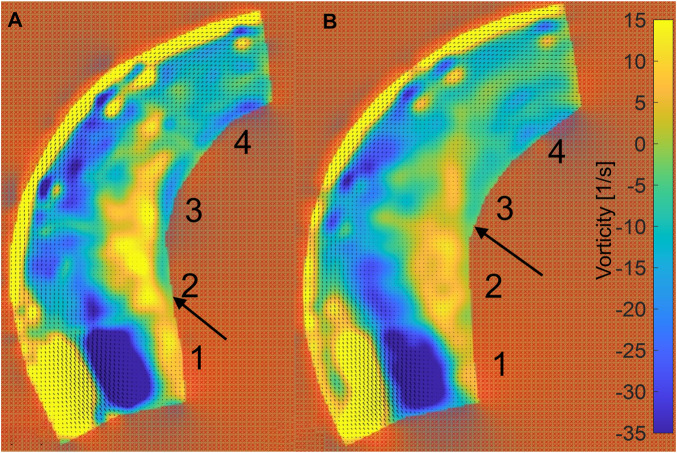
Vorticity contour of ascending aortic flow field at pulsating pressure. **(A)** The contour for the inlet at low pressure (blue line in Figure 5C). **(B)** The contour for the inlet at high pressure (red line in Figure 5C).

The PIV results are given for two situations: low pressures in the range of 15.73–16.92 kPa and high pressures in the range of 22.44–23.55 kPa, as shown in Figure 5C. After analyzing the 4,000 pairs of photos (for a duration of 1.6 s, as the camera took photographs at a rate of 2,500 frames per second), as before, we averaged the velocity field over the 1.6 s. The velocity contour and velocity vector field are shown in Figure 11. Additionally, the vorticity contour is shown in Figure 12.

Figure 11 shows that the inner curve is dominated by the low-velocity field (velocity <0.1 m/s), while the velocity is high at the outer curve. Interestingly, the low-velocity region is larger after a pressure increase. According to the velocity vector field, the downstream workflow is near the outside, while there is more rotation in the low-velocity region.

Figure 12 shows the vorticity in the phantom. The edge of the outside vorticity was ignored because the vorticity is clearly influenced by the 10-mm marked lines. The incident region shows a distinct low vorticity, which is caused by the normally incoming flow. Meanwhile, the inside of the phantom shows a high level of vorticity (vorticity >5 s^−1^). After the increase in pressure, the high vorticity region is maintained in area 3 near the wall on the inner side, while Area 2 is at low pressure, as indicated by the arrows in Figure 12.

## 4 Discussion

*In vitro* experiments are an excellent way of investigating the complex mechanical processes in the aorta; they avoid the strict ethical issues of *in vivo* experiments, as well as the sophisticated implementation of fluid–structure interaction (FSI) simulations and the associated large computational cost. In this study, the main purpose was to investigate deformations of the aorta after an increase in terminal resistance and aortic hemodynamics using PIV, as well as revealing the potential for injury of the aorta after an increase in terminal resistance.

### 4.1 Morphological changes of the aorta after an increase in pressure

The results shown in Figure 8 reveal that with an increase in pressure, significant elongation appeared in the aortic phantom in areas 2 and 3, corresponding to the middle of the ascending aorta, and area 7, which is the beginning of the descending aorta. The two areas experienced considerably more stretching deformation after an increase in impedance than other areas. In particular, these areas become elongated sooner. Comparison of the elongation of the outside contour, shown in Figure 9, with the 10-mm marked lines shown in Figure 8 showed that there was unequal elongation over the surface of the aorta. Finally, the inside contour deformed by another mode, in which the aortic wall of the aortic arch and descending aorta contracts, indicating that the aortic wall was compressed, as shown in Figure 9. However, the dilation of the aorta was consistent throughout the whole aortic phantom, showing that a certain degree of dilation occured at all eight cross-sections.

The anisotropic character of the aortic wall is determined by its structure. The percentage of elastic fibers in the aortic wall is much smaller in the axial direction than in the circumferential direction (Sokolis et al., 2006; Hoefer et al., 2013); this might lead to a high possibility of injury when the vessel is exposed to axial stretching deformation. By combining our morphological results we can infer that there is a greater possibility of injury in a real aorta as a result of axial stretching after pressure increases at areas 2 and 3. Moreover, because of the positive Poisson’s ratio of silicone and the aortic wall, elongation and dilation of the aorta leads to qualitative thinning of the aortic walls, especially in the ascending aorta. This situation means that the aortic wall is under greater stress, but this stress can be decreased by reducing the aortic diameter and thickening the aortic wall (Hardikar et al., 2020). At the level of bioregulation, experiments in hypertensive rats (Intengan and Schiffrin, 2001) have demonstrated enhanced arterial remodeling.

It is helpful to reveal potential mechanisms by which tearing occurs at certain positions of the aorta using the morphological characteristics of the aortic phantom. There have been reports of tearing at the anatomical positions of areas 2 and 3 (Gao et al., 2006; Poullis et al., 2008; Taguchi et al., 2014). We also discovered that the 50% tearing position of the type A aortic dissection patients in our previous study (Chi et al., 2017) is in the same anatomical position as our experimental results. Additionally, Weiss et al. (Weiss et al., 2012) studied the relationship between the position of a primary entry tear and its outcome in a sample of 52 patients with type B aortic dissection, and found that, in 48% of the patients studied, the primary entry tear was located at the convexity of the distal aortic arch, which is the same region as the outside of areas 5 and 6. At the same time, in 52% of the patients studied, the primary entry tear was located at the concavity of the distal aortic arch, which is the same region as the inside of areas 5 and 6. Weiss et al. (Weiss et al., 2012) identified two types of tear characteristics that predispose the development of complicated acute type B aortic dissection. On the one hand, if the tear was on the inside contour, near areas 5 and 6, rapid disease progression was experienced by 89% of affected patients. In comparison, when the tear occurred on the outer contour, rapid tear progression occurred in only 20% of patients with aortic dissection. On the other hand, the closer the tear is to the left subclavian aorta, the greater the likelihood that complicated acute type B aortic dissection will be present or develop. The results of our experiment present the circumferential distension (Figure 10) and axial compression (Figure 9) of the aortic wall during a pressure increase on the inside contours of areas 5 and 6. This circumferential distension might lead to a greater tendency for the intima and media layers near the tear to detach from the adventitia layer, while axial compression might lead to shear strain in the aortic wall near the tear, where it adheres to the tear (Sherifova and Holzapfel, 2019), thus making a tear more likely to form or develop.

### 4.2 Change of hemodynamics in the aorta due to an increase in pressure

According to the morphological alteration of the aortic phantom, there is greater elongation and dilation at the ascending aorta than at the descending aorta. There will be a consequent change in the intravascular hemodynamics at the ascending aorta. Here, a PIV visualization study was performed on a portion of the ascending aorta irradiated by a laser. The flow field data were acquired for a two-dimensional plane of the three-dimensional flow field within the ascending aorta, as shown in Figure 5B. After post-processing in PIVlab (Thielicke and Sonntag, 2021), the contours of the velocity and vorticity are shown in Figures 11, 12, respectively. Although the flow field of the outside is not accurate due to the marked lines, the inside is not influenced by the marked lines. The hemodynamics show two characteristics. First, the flow field presents an increasing area of low velocity, corresponding to the region of high vorticity shown in Figure 12, inside the ascending aorta at a high pulsation pressure, as shown by the red dashed line in Figure 11. Second, the direction of inlet flow changes with an increase in the terminal impedance. The two aspects caused by the morphological change after the impedance increase, namely, dilation of the aortic phantom and a change in the direction of the inlet flow, result in an increased area of the vortex region. In our previous numerical study, the dilation and elongation of the ascending aorta result in an increase of the vortex region and this might cause an abnormal oscillatory shear index at the inside surface of the ascending aorta (Chi et al., 2021b). Therefore, it can be concluded that areas 1 to 3 exhibit worse hemodynamics due to the increase of the vortex region shown in Figure 11B. Our results suggest that there is a risk factor of tearing as a result of hypertension or a pressure increase caused by a physical impact.

To investigate the morphology and hemodynamics in the aorta resulting from FSI, the *in vitro* experiment using PIV is an excellent approach, with the advantage of the lower computational cost and avoiding the technical bottleneck of numerical FSI. The boundary conditions of the experiments are achieved in two ways. One involves increasing the pressure in the tube while maintaining a constant flow rate. The other involves increasing the magnitude of the pressure waveform by keeping the period and frequency constant. If rigid walls had been used in the experiment, impedance increases of different magnitudes would theoretically only cause changes in the flow field values. However, the impedance increases under the effect of FSI resulted in morphological changes, such as dilation and elongation of the ascending aorta, which again caused a change in the flow field. The PIV results present the alteration of aortic morphology and the hemodynamics induced by the increase of pressure simultaneously, suggesting a sophisticated FSI phenomenon.

Although the sudden increase in pressure directly leads to the deformation of the blood vessel, which further leads to a worsened stress state of the vessel wall, the wall shear stress on the interface of the aortic wall is a long-term determinant of the aortic diameter. Using PIV, we found that the hypertension-induced morphological changes, which in turn alter hemodynamic factors, are probably long-term effects of vascular remodeling.

### 4.3 Limitations and future work

In this study, *in vitro* experiments were conducted to investigate aortic morphology and hemodynamics using PIV. However, there remain some limitations. First, the mechanical property of the silicone phantom is not exactly the same as the natural aortic wall; this could be improved in future work by selecting an improved silicone material and refining the brush-spin-coating method (Chi et al., 2021a). Then, only the aorta is concerned in this study; thus, a simplified aortic phantom without branches was used. However, the structure of the branch might also tear due to its shape (Chi et al., 2017; Chi et al., 2021b). Therefore, the effect of the branch on aortic morphology and its relationship to the tearing should be investigated in future studies. Meanwhile, we lengthened the inlet and outlet as the auxiliary flow part when making the phantom. Thus, the influence of the fixed inlet and outlet on the experiment can be avoided. However, the physiological aorta is in the complex tissue support (Moireau et al., 2012); this improvement can be made in the future.

Our PIV study presents the hemodynamics *in vitro*. The vortex region is presented clearly. However, only a simple sine pressure wave was used in the PIV experiment. The input waveform could be refined to be closer to a physiological waveform in future studies. In addition, only a two-dimensional plane at the ascending aorta was used to present the PIV result to assess the three-dimensional flow field. In subsequent studies, measurements might be made in more planes, for instance, the vertical plane, to approach a three-dimensional flow field. Moreover, the descending aorta should also be investigated because the tears will also occur here for type B aortic dissection.

## 5 Conclusion

In this study, first, considering the refractive index and mechanical properties of silicone, we manufactured a phantom aorta. Then, *in vitro* experiments were conducted. The *in vitro* experiments provide an insight into changes in the aortic morphology and the hemodynamics when the pressure is increased. The use of a silicone phantom is extremely suitable in the study of hemodynamics and aortic morphology, as it avoids the difficulties inherent in *vivo* experiments and numerical FSI simulations. Our study suggests that the elongation of the aorta in response to elevated pressure is a morphological characteristic; the increased pressure expands the middle position of the ascending aorta, resulting in a larger possible accumulative region of aortic injury because of the underlying hemodynamics. According to this study, increasing terminal impedance led to a worsened state of the inner side aortic wall at the ascending aorta; this might be a risk factor for tearing and should be investigated more closely.

## Data Availability

The raw data supporting the conclusion of this article will be made available by the authors, without undue reservation.
